# Are reprogrammed cells a useful tool for studying dopamine dysfunction in psychotic disorders? A review of the current evidence

**DOI:** 10.1111/ejn.13418

**Published:** 2016-10-19

**Authors:** Ulrich Sauerzopf, Roberto Sacco, Gaia Novarino, Marco Niello, Ana Weidenauer, Nicole Praschak‐Rieder, Harald Sitte, Matthäus Willeit

**Affiliations:** ^1^ Department of Psychiatry and Psychotherapy Medical University of Vienna Währinger Gürtel 18‐20 1090 Vienna Austria; ^2^ Institute of Science and Technology Austria Klosterneuburg Austria; ^3^ Institute of Pharmacology Medical University of Vienna Vienna Austria

**Keywords:** bipolar disorder, dopamine, induced neuron, induced pluripotent stem cell, neuronal progenitor cell, schizophrenia

## Abstract

Since 2006, reprogrammed cells have increasingly been used as a biomedical research technique in addition to neuro‐psychiatric methods. These rapidly evolving techniques allow for the generation of neuronal sub‐populations, and have sparked interest not only in monogenetic neuro‐psychiatric diseases, but also in poly‐genetic and poly‐aetiological disorders such as schizophrenia (SCZ) and bipolar disorder (BPD). This review provides a summary of 19 publications on reprogrammed adult somatic cells derived from patients with SCZ, and five publications using this technique in patients with BPD. As both disorders are complex and heterogeneous, there is a plurality of hypotheses to be tested *in vitro*. In SCZ, data on alterations of dopaminergic transmission *in vitro* are sparse*,* despite the great explanatory power of the so‐called DA hypothesis of SCZ. Some findings correspond to perturbations of cell energy metabolism, and observations in reprogrammed cells suggest neuro‐developmental alterations. Some studies also report on the efficacy of medicinal compounds to revert alterations observed in cellular models. However, due to the paucity of replication studies, no comprehensive conclusions can be drawn from studies using reprogrammed cells at the present time. In the future, findings from cell culture methods need to be integrated with clinical, epidemiological, pharmacological and imaging data in order to generate a more comprehensive picture of SCZ and BPD.

## Introduction

Reprogrammed cells offer a window into a previously inaccessible sphere of neuro‐psychiatric disorders filling a gap between *in vivo* observations of clinical cases and genetic analysis. So far, research on the pathogenesis of psychiatric disorders on a cellular level has been notoriously difficult due to the lack of proper tissue models for testing hypotheses or medicinal compounds. Tissue sampling has given some insight into differential functioning of cells between healthy subjects and patients, but cannot avoid the doubt inherent to drawing conclusions from one tissue to another. No specimen can be routinely taken from the central nervous system. Animal models have unequivocally been helpful in testing compounds and generating hypotheses on the pathogenesis of various psychiatric disorders. However, as psychiatric disorders are complex and multifactorial, it is unlikely that a single animal model will be able to fully reproduce their pathophysiology (Jones *et al*., 2011). Moreover, with somatic disease models, we cannot assess whether the laboratory animal does actually display the psychiatric symptoms we are trying to investigate. While affective disorders comprise many symptoms directly linked to behaviour, all of the Schneiderian first‐rank symptoms traditionally used to define core schizophrenia (SCZ) belong to the realm of subjective experience (Schneider, 1957; Tandon *et al*., 2009). We cannot ask a mouse whether it experiences thought broadcasting or hears voices. Sampling of post mortem tissue avoids uncertainty of pathology as it is possible to select patients based on their clinical history. Those analyses are nevertheless confounded by lack of information on earlier episodes, often lifelong medical treatment, post mortem decay, and by death itself, which, among other things, destroys ion gradients and alters tissue functioning and composition.

Since the publication of a robust method for the generation of induced pluripotent stem cells (iPSC) by forced expression of four transcriptional factors (*Klf4*,* Oct4*,* Sox2* and *c‐Myc*) by Takahashi & Yamanaka, in (2006) the road to *in vitro* assays for neuronal cells was paved (Takahashi & Yamanaka, 2006; Takahashi *et al*., 2007). Those pluripotent cells can be differentiated into all three germ layers, so that easily accessible cells such as fibroblasts or hematopoietic cells can be used to yield neuronal progenitor cells and neurons. Inspired by the successful reprogramming of cells with transcriptional factors, Vierbuchen *et al*. published a method for directly converting mature somatic cells to functional neurons by employing the factors *Ascl1*,* Brn2* and *Myt1l* in 2010. Those cells showed signs of mature neurons including neuronal membrane potentials and the formation of functional synapses displaying short‐term plasticity (Vierbuchen *et al*., 2010). A host of reprogramming techniques, either for direct transformation to a target cell, or to a pluripotent state, has been published since.

Today, the notion of altered dopamine (DA) neurotransmission in SCZ is no longer a hypothesis but supported by a wealth of evidence derived from neuroimaging and genetic studies. Results from the two research fields, found with a diverse array of methods, converge on disturbances in pre‐synaptic DA transmission as a key element in the pathogenesis of SCZ. Imaging studies show increased DA uptake and storage in the basal ganglia, and an enhanced release of DA into the extracellular space in patients with SCZ. Large‐scale genetic studies show an association of SCZ with gene loci coding for DA receptors and proteins involved in synaptic transmission. However, integrating these findings to understanding the impact of genetic and environmental risk factors for SCZ at a cellular level emerges as a one of the key challenges on our way to new drug targets. Research in cultured neurons has the potential to help closing this gap. Instead of just antagonizing DA D_2/3_ receptors at the postsynaptic membrane, alternative ways for restoring DA transmission at the pre‐synaptic level will hopefully lead to more efficient and better tolerated ways to treat psychotic symptoms in major psychiatric disorders.

In this review, we the summarize findings from cell culture models of SCZ and bipolar disorder (BPD) and attempt to put these findings into the context of current hypotheses. We will first summarize findings from cell culture models suggesting altered synaptic transmission, then we describe evidence focussing on altered cell maturation and early neurodevelopment, and then we report on literature focussing on cellular energy metabolism. An overview on details and limitations of current reprogramming methods is found in the appended ‘Techniques of cell reprogramming’ section.

## Schizophrenia

Schizophrenia is a psychiatric disorder that affects roughly 1% of the population worldwide (Saha *et al*., 2005). The clinical presentation of the disorder is highly heterogeneous, commonly comprising positive symptoms such as delusions or hallucinations, negative symptoms such as poverty of speech or motivational disturbance, cognitive symptoms such as impaired working memory or executive malfunctioning, and affective symptoms such as depressed or hypomanic mood (van Os & Kapur, 2009). A single patient can display a plurality of symptoms, so that two patients, each being diagnosed with the same disorder, need not share many psychopathological traits.

Twin studies have shown that SCZ has a high heritability of up to 90% (Sullivan *et al*., 2003), making it a natural target for cell‐based assays. There is a limited number of loci of relatively high impact such as Disrupted in schizophrenia 1 (DISC1), first characterized as a risk factor in a Scottish family (Millar *et al*., 2000), or the deletion 22q11.2, which causes the DiGeorge syndrome (Lindsay *et al*., 1995). A vast number of low impact risk loci have been identified over time, each contributing only marginally to the manifestation of the disorder in an individual (Duan, 2015). Yet, a significant proportion of heritability found in twin studies remains unaccounted for (Manolio *et al*., 2009; Eichler *et al*., 2010). A significant proportion of SCZ‐associated risk loci were significantly enriched by enhancers not only in neuronal, but also in peripheral tissue (Psychiatric Genetics Consortium, 2014). Contributions to the development of SCZ by immunological effectors are supported by the recent observation of the involvement of complement component 4 (C4) in synaptic pruning (Sekar *et al*., 2016). Significant enrichment was also found in skeletal muscle and pancreatic islands, which implies some involvement of non‐neuronal tissues and is in line with findings of disturbed metabolism in SCZ (Psychiatric Genetics Consortium, 2014).

In 2011, Chiang *et al*. were the first to transform cells derived from patients suffering from SCZ to pluripotent stem cells utilizing an integration‐free plasmid vector (Chiang *et al*., 2011). Since this pioneering work, a host of studies has dealt with patient‐derived reprogrammed cells. Most frequently, skin fibroblasts were harvested by punch biopsy and consequently transformed into iPSCs and differentiated into neuronal progenitor cells (NPCs) and a neuron‐like state. Robicsek *et al*. (2013) were, to the best of our knowledge, the only ones to prefer hair follicle keratinocytes over fibroblasts for their more efficient reprogramming, availability and ectodermal identity. Apart from low numbers of independent donors, a tendency to recruit patients displaying strong signs of pronounced genetic background such as high‐risk mutations, high‐incidence families and early childhood onset of the disorder can be noted. Meir Jacobs’ review from 2015 gives an overview about possibilities and limitations of iPSC‐based studies in the field of SCZ (Jacobs, 2015). A technical review is provided by Duan, (2015).

## Bipolar disorder

Bipolar disorder (BPD) is characterized by recurrent episodes of depression and mania. Mania in particular is often accompanied by psychotic states. Similar to SCZ, there is a high genetic contribution to the disorder. In twin studies, heritability between 59 and 87% was noted (Smoller & Finn, 2003). Among the available treatment modalities, Lithium (Li+) displays the greatest efficacy and a robust anti‐suicidal effect (Cipriani *et al*., 2013). Li+ interacts with cyclic adenosine mono‐phosphate (cAMP) response element‐binding (CREB) protein activity, the membrane potential of neurons via the Na+/K+ ATPase, monoamine neurotransmitters, second messenger cascades, glycogen synthase kinase (GSK) activity and transcriptional factors (Alda, 2015). In the publications concerning reprogrammed cell models derived from BPD patients reviewed here, response to Li+ responsiveness plays a prominent role.

For an overview on patient‐derived reprogrammed cell studies in the field of SCZ and BPD, see Tables 1 and 2.

**Table 1:** Summary of the literature on findings in reprogrammed neuron‐like cells derived from patients with schizophrenia and schizoaffective disorder

Publication	Culture model	Cases	Cells	Summary of findings
Chiang *et al*. (2011)	Fibroblasts → iPSCs Episomal vector (EBV based) Plasmids (pEP4 EO2S ET2K, pEP4 EO2S EN2L, pEP4 EO2S EM2K)	Two cases chronic SCZ, *DISC1* mutation, siblings	No Differentiation past iPSC	Proof of concept, for iPSC generation from SCZ patients. First SCZ‐derived iPSC line
Brennand *et al*. (2011)	Fibroblasts → iPSCs Tetracycline induceable Lentiviral vector (*OCT4*,* SOX2*,* KLF4*,* c‐MYC*,* LIN28*) → NPCs → iNeurons	Five cases (three SCZ, one SZA and drug abuse, one Schizoid personality and anorexia nervosa): Four in high‐incidence families One childhood onset Coriell Cell Repository GM02038 GM01792 GM01835 GM02497 GM02503 Six age‐ and ancestry‐matched controls GM02937 GM03440 GM03651 GM04506 AG09319 AG09429	30% GAD65/67+ 10% TH+ 60% VGLUT1+	Cell‐connectivity ↓ in SCZ Neurite outgrowth ↓ in SCZ PSD‐95 ↓ in SCZ Altered cAMP/WNT signalling Loxapine rescues alterations 42 Genes affected by CNV of those only *CSMD1, MYH1, MYH4* showed > 1.3 fold expression difference 596 Genes differently expressed (> 1.3 fold) 25% of those connected to SCZ
Pedrosa *et al*. (2011)	Fibroblasts → iPSC Retroviral vector driven by long terminal repeat (*OCT4*,* SOX2*,* KLF4*,* c‐MYC)* → NPCs → iNeurons Neuronal differentiation medium, WNT3A to support glutamatergic cell fate	Three cases: One del(22q11.2) SCZ One childhood onset One familial case Two latter cases Coriell Cell Repository GM02039 GM02497 Two healthy subjects	Most cells are glutamatergic and express VGLUT1, GRIK3, AMPA2, GRIA2 Weak expression of GAD1 and TH	In del(22q11.2), decline of *OCT4* and *NANOG* expression was markedly delayed *SMARCA2, JARID2, MYT1L* and *NPAS3* all of which have been related to SCZ are expressed > 2 fold differently > 2 fold differences in the expression in SCZ‐relevant genes *CHGB*,* SLC25A27*,* C4A*,* CHL1* and *CTNNA2. NRXN3* was expressed at > 3 fold difference 1207 Genes expr. diff. The most significant GO cluster comprised ‘neurogenesis’, ‘neuronal differentiation’, ‘axon guidance’, and ‘adhesion’
Paulsen Bda *et al*. (2012)	Fibroblasts → iPSCs pMX‐based retroviral vector (*OCT4*,* SOX2*,* KLF4*,* c‐MYC)* → NPCs Retinoic acid and FGF2	One case, clozapine‐resistant SCZ One age‐matched healthy control	Differentiation to NPCs AADC, DAT, TH, ChAT and LMX1B were detected	Extramitochondrial O2 consumption ↑ in SCZ‐derived NPCs but not in iPSC or fibroblasts Level of reactive oxygen species ↑ in SCZ‐derived NPCs Valproic acid reverts reactive oxygen species level No changes in ATP‐coupled respiration
Robicsek *et al*. (2013)	Hair follicle keratinocytes → iPSC STEMCCA Lentivirus reprogramming kit → NPCs → iNeurons Dual SMAD inhibition for DA neurons No use of WNT3A or retinoic acid for GLUT differentiation	Three cases paranoid SCZ, clozapine treated Two healthy subjects	Differentiation to DA and GLUT neurons	SCZ‐derived iDA NPCs show higher *PAX6* and reduced Nestin staining. Larger cell area. Conversion to iDAs is incomplete in SCZ cells. None express DAT, little release of dopamine and very few b3‐Tubulin+/TH+ cells. Outgrowth ↓ In Glut differentiation SCZ‐derived cells express no *Tbr1; synapsin1* and *PSD‐95* ↓ indicative of fewer synaptic contacts. Mitochondiral dysfunction described in SCZ‐derived cells including keratinocytes
Hook *et al*. (2014)	Fibroblasts → iPSCs Tetracycline inducible lentiviral vector (*OCT4*,* SOX2*,* KLF4*,* c‐MYC*,* LIN28*) → NPCs → iNeurons BDNF, GDNF, dibutryl‐cAMP and ascorbic acid	Three cases, SCZ One childhood onset Coriell Cell Repository GM01792 GM02038 GM2497 Three controls American Type Culture Collection AG09429 Coriell Cell Repository GM04506 One neonatal foreskin	Mixed population	> 2 fold number TH+ neurons derived from SCZ than healthy subjects’ iPSCs Large differenced between patients No increased transcription of TH Elevated release of dopamine, norepinephrine and epinephrine in SCZ‐derived iPSC neurons
Yu *et al*. (2014)	Fibroblasts → iPSC Tetracycline induceable Lentiviral vector (*OCT4*,* SOX2*,* KLF4*,* c‐MYC*,* LIN28*) → NPCs DKK1 Cyclopamine, Noggin → iNeurons WNT3A, BDNF	Four SCZ cases, derived from Brennand *et al*. (2011) Coriell Cell Repository GM02038 GM01792 GM01835 GM02497	> 85% vGLUT+ < 15% GABA+ Hippocampal DG granule neurons	Lower fraction of active neurons in SCZ Delayed conversion of NPCs to DG granule neurons in SCZ Reduced spontaneous neurotransmitter release and spontaneous excitatory post‐synaptic currents in SCZ
Yoon *et al*. (2014)	Fibroblasts → iPSC Sendai virus or episomal vector in concordance with Chiang *et al*. (2011) → NPCs → iNeurons	Five cases Three del(15q11.2) no clinical information Two DISC1 chronic SCZ (identical Chiang *et al*. (2011)) Three healthy subjects	Differentiation to NPCs No information on dcell identity	*CYFIP1* (a gene involved in 15q11.2 CNVs) and Wave signalling mediators ACTR2/Arp2 SNPs do while not causing risk for SCZ individually, interact epistatically confer significant risk for SCZ. This may be due to derogation of apical polarity and adherens junctions in affected neurons
Wen *et al*. (2014)	Fibroblasts → iPSCs Episomal vector (EBV based) Plasmids(pEP4 EO2S ET2K, pEP4 EO2S EN2L, pEP4 EO2S EM2K) → NPCs → iNeurons	Two cases from one family One SCZ One major depression Pedigree H, frameshift mutation in *DISC1* Three healthy subjects (One neonatal foreskin, two not affected relatives)	Forebrain identity 90% of neurons expressed *VGLUT1* or α*‐GAMKII* few *GAD6+7*,* VGAT+* or TH+	Impaired synaptic transmission in *DISC1* mutation carriers. Defect in depolarization induced vesicle release. 1–2 weeks post‐conversion *DISC1* carriers display larger soma and dentritic length. A large number of genes is differentially expressed at 4 weeks. Those are linked to synaptic transmission, dentritic spines and CNS development. 89 genes linked to mental Disorders
Hashimoto‐Torii *et al*. (2014)	Fibroblasts → iPSCs Tetracycline induceable Lentiviral vector (*OCT4*,* SOX2*,* KLF4*,* c‐MYC*,* LIN28*) → NPCs → iNeurons	Four cases, No further characterization Five controls	No differentiation past NPCs No information on cell identity	Following environmental challenges with methyl mercury and ethanol, NPCs derived from patients display sig. larger variability in HSP70 expression than healthy subjects. Means of HSP70 expr. are unchanged. GAPDH expression shows no difference
Paulsen Bda *et al*. (2014)	Fibroblasts → iPSCs pMX‐based retroviral vector (*OCT4*,* SOX2*,* KLF4*,* c‐MYC)* → NPCs Retinoic acid and FGF2	One clozapine‐resistant patient SCZ Two healthy subjects	No differentiation past NPCs	Significantly more Zn+ and K+ in SCZ‐derived NPC (but not iPSC) clones. Valproate reduced Zn+ and K+ levels to normal without affecting other trace elements as measured by syncrotron radiation x‐ray microflurescence spectrometry
Topol *et al*. (2015)	Fibroblasts → iPSC Tetracycline induceable lentiviruse vectors (*OCT4*,* SOX2*,* KLF4*,* c‐MYC*,* LIN28*) → NPCs → iNeurons	Four cases with SCZ Coriell Cell Repository GM02038 GM01792 GM01835 GM02497 Six healthy subjects	Study in NPCs forebrain identity	Significant over‐expression of translation‐related proteins with globally increased protein levels. Overexpression of protein clusters related to ribosome/RNA binding, nucleosome, chromatin, nucleocytoplasmatic and transport. Increased Translation is observed in NPCs but not in iPSCs. Primary Fibroblasts show a trend towards increased synthesis. The findings cannot be explained by cell size. No link between aberrant migration and protein over expression could be established
Brennand *et al*. (2015)	Fibroblasts → iPSCs Tetracycline induceable Lentiviral vector (*OCT4*,* SOX2*,* KLF4*,* c‐MYC*,* LIN28*) → NPCs → iNeurons	Four cases with three SCZ 1 SZA Two patients are siblings Coriell Cell Repository GM02038 GM01792 GM01835 GM02497 Six healthy subjects Coriell Cell Repository GM03440 GM03651 GM04506 American Type Culture Collection AG09319 AG09329 One neonatal foreskin CRL‐2522	Forebrain NPCs and early neurons	NPC and early neuron gene expression resembles first trimester forebrain neurons. SCZ and healthy subjects display similar gene expression and spatial and temporal identity. *NCAM1*,* NLG1*,* NRXN1* and *NRXN3* ↓ in SCZ 481 genes expressed at > 1.3 fold difference between SCZ and healthy subjects in NPCs; differences are conserved in older stages. Aberrant cell migration in SCZ cells (no effect of loxapine or clozapine) Morphological difference in mitochondria Increased oxidative stress in SCZ
Passeri *et al*. (2015)	Fibroblast direct conversion to iNeurons Lentiviral vector (*ASCL1*,* POU3F2*,* MYT1L*) novel conversion media	Four cases, childhood onset 2 del(22q11.2) 1 del(16p11.2) 1del(16p11.2)dupl.(22q13.3) Five healthy subjects	Mostly glutamatergic	del(16p11.2) is associated with significantly higher rate of conversion to neuron‐like cells
D'Aiuto *et al*. (2015)	Fibroblasts → iPSC → NPCs → iNeurons	One case SCZ Line 5404 One control neonatal foreskin HFF1‐S	Differentiation to glutamatergic neurons	Herpes simplex virus I can establish quiescent infection in iPSC‐derived neurons and alters gene expression in cognition relevant pathways. No information provided on differences regarding diagnosis
Zhao *et al*. (2015)	Fibroblasts → iPSC Nucleofection plasmids (*OCT4*,* SOX2*,* KLF4*,* L‐MYC*,* LIN28* and p53 shRNA) → NPCs → iNeurons	Six cases 4 del(22q11.2) (1 SCZ, 3 SZA) Two childhood onset SCZ Six healthy subjects	GABAergic glutamatergic mixed population	45 miRNAs expressed differently according to diagnosis. Of 6 miRNAs with genome wide sign. down regulated in del(22q11.2) cells; 4 map to affected region (miRNA‐1306‐3p, miR‐1286, miRNA‐1306‐5p and miRNA‐185‐5p) and 2 do not (miRNA‐3175, miRNA‐3158‐3p) miRNA‐34 family miRNAs showed marked increase but did not reach genome wide sign. miRNA‐4449, miRNA‐146b‐3p, and miRNA‐23a‐5p showed the greatest increase in expression. No upregulated miRNAs reached genome wide sign
Lee *et al*. (2015)	Cohort 2: Fibroblasts → iPSCs Sendai viral vector → NPCs → Neurons Dual SMAD inhibition Lentiviral vector (*Ngn2* induction) to yield glutamatergic neurons	One case (offspring) SZA heterozygous *CNTNAP2* deletion Seven healthy subjects (father) heterozygous *CNTNAP2* deletion (mother) no mutation Five unrelated healthy subjects	Forebrain identity and glutamatergic neurons late oligodendrocyte precursor cells (15% O4+/MBP+ mature oligodendrocytes) as well as ~15% astrocytes and ~20% neurons	Increased expression of the full length transcript in a phenotype‐specific way. In the SZA patient in 6‐weeks old neurons. Not affected father displayed increased transcription only in NPCs. NPC migration was significantly reduced in the SZA case, but not in the healthy carrier (father). In SZA (offspring) oligodendrocyte precursor cells predominantly the mutated allele of *CNTNAP2* was expressed, whereas the father primarily expressed the wild‐type allele
Murai *et al*. (2016)	Fibroblasts → iPSCs Lentiviral vector → NPCs	Two cases SCZ Both from pedigree H with *DISC1* frameshift mutation Three healthy subjects	No differentiation past NSC	miR‐219 expression is elevated in DISC1 carrier NSCs. miR‐219 acts downstream of *TLX* and leads to reduced NSC proliferation
Topol *et al*. (2016)	Cohort 1: iPSCs → NPCs → Neurons Tetracycline induceable Lentiviral vector (*OCT4*,* SOX2*,* KLF4*,* c‐MYC*,* LIN28*) Cohort 2: Fibroblasts → iPSCs Sendai viral vector → NPCs → Neurons Dual SMAD inhibition	Cohort 1: Four SCZ cases, six controls Cohort 2: 10 childhood onset SCZ cases 10 not related healthy subjects	NPCs and early neurons, forebrain identity	SCZ NPCs display reduced miR‐9 levels. A subset (~50%) of SCZ are below 25% quantile of healthy subjects’ NPCs. Perturbed miRNA‐9 levels can be observed in NPCs and early neurons but not after prolonged (6 weeks) maturation miRNA‐9 is correlated with impaired radial migration of NPCs in SCZ. Overexpression of miRNA‐9 rescues migration phenotype. Non‐genomic as well as genomic causes might affect altered miRNA‐9 Altered miRNA‐9 expression affects multiple genes on transcript and protein level

iPSC, induced pluripotent stem cells; SCZ, schizophrenia; NPC, neuronal progenitor cell; SZA, schizoaffective disorder; CNV, copy number variation; iNeuron: induced neuron‐like cell; iDA, induced dopaminergic neuron.

**Table 2:** Summary of the literature on findings in reprogrammed neuron‐like cells derived from patients with bipolar disorder

Publication	Culture model	Cases	Cells	Summary of findings
Chen *et al*. (2014)	Fibroblasts → iPSC Retroviral vector (*OCT4*,* SOX2*,* KLF4, c‐MYC*) iPSC → NPC → iNeurons Embryoid body medium and neuron induction medium	Three cases BPD type 1 2 Li+ responders, one non‐responder three age‐matched healthy controls	Forebrain phenotype VGlut+ pyramidal‐shaped neurons, GABA+ interneurons, scattered serotonergic and dopaminergic neurons	2547 genes expression diff. between BPD/healthy subjects’ iPSC 186 genes expression ↑ BPD (> 1.5 fold) in iPSC 46 genes expression ↓ BPD (> 1.5 fold) in iPSC 1312 genes expression diff. between BPD/healthy subjects’ iN 140 genes expression ↑ BPD (> 1.5 fold) in iN 46 genes expression ↓ BPD (> 1.5 fold) in iN BPD‐derived neurons show higher expression of *DICER1*,* NKX2‐1* and *PAX2* but lower expression of *EMX2* and *HOXA1,6,7* Li+ pre‐treatment reduces Ca++ transient and wave amplitude in BPD‐derived iNs compared to healthy subject derived iNs
Wang *et al*. (2014)	Fibroblasts direct conversion Lentiviral vector (miRNA‐9/9*‐123, *NEUROD2*,* ASCL1*,* MYT1L*)	Twelve cases BPD type 1 and history of Li+ treatment Six responders Six non‐responders Six healthy subjects	Heterogeneous cell population, no capability to produce action potentials without further differentiation and expressing neuronal as well as stem‐cell markers	Cells derived from Li+ responders show greater adhesion than cells from Li+ non‐responders as measured by ∆PWV (Peak Wave Length) in a BIND scanner
Madison *et al*. (2015)	Fibroblasts → iPSC Retroviral vector (vector (*OCT4*,* SOX2*,* KLF4, c‐MYC*)) iPSC → NPC → iNeurons NPC medium, neurobasal medium	Two offspring (BPD Type 1) GM05224 GM05225 Coriell Cell Repository Two parents (healthy) GM08330 GM08329	Mixed population	Neurons could be generated from all healthy subjects’ iPSC but only 2/6 BPD iPSC lines. NPCs could be generated from all lines, but not propagated beyond 4–5 passages. No expression of *PAX6* Bromodeoxyuridine staining (proliferation) ↓ in BPD NPCs BPD neurons show ↓↓ viability after 2 weeks of differentiation; hardly any neurons were found after 6 weeks In iPSCs and fibroblasts, hardly any difference between BPD and healthy subjects In NPCs, 18 genes > 1.5 fold expression
Mertens et al. (2015)	Fibroblasts → iPSC → NPCs Cyto‐Tune Sendai reprogramming kit (commercial) → NPCs DKK1 Cyclopamine, Noggin → iNeurons WNT3A, BDNF	Six cases, BPD Type 1 Recruited during a prospective trial for Li+ monotherapy (three Li+ responders, three Li+ non‐responders) Four healthy subjects	Hippocampal dentate gyrus (DG) granule cell‐like neurons > 80% VGLUT1+ 2–7% GABAergic (characterized by GABA release)	Expression of mitochondrial genes in BPD ↑ Overall smaller mitochondria mitochondrial function in JC‐1 Assay in BPD ↑ Increased excitability in BPD iPSC neurons. Altered action potential frequency, Ca++ signalling PKA/C signalling. In Li+ responders, action potential frequency was attenuated by Li+ but not in non‐responders. Li+ alters gene expression differently in responders and non‐responders and seems to rescue disease relevant genes. Li+ increases mitochondria size. Electrophysiological alterations in non‐responders were attenuated by lamotrigine
Bavamian *et al*. (2015)	iPSC → NPCs Retroviral vector (vector (*OCT4*,* SOX2*,* KLF4, c‐MYC*)) NPCs→iNeurons In NPC and neuronal differentiation media Fibroblasts → iNeuron: direct conversion from fibroblasts Lentiviral vector (miRNA‐9/9*‐123, *NEUROD2*,* ASCL1*,* MYT1L*)	iPSC: I case BPD type 1 Coriell Cell Repository GM05224 and one healthy subject GM08330 iN five cases BPD type 1	Not stated	miRNA‐34a ↑ in BPD patient cerebellum slices (without Li+ treatment) as well as in both cell models 25 putative miR‐34a targets were identified. *ANK3*,* CACNA1C*,* CACNB3*,* ODZ4*,* DDN*, and *KLC2* overlap with BPD GWAS loci. miRNA‐34a targets were downregulated with increased expression of the miRNA in NPCs and early neurons derived from a BPD patient. *ANK3* and *CACNB3* were up regulated as miRNA‐34a was blocked in healthy subject and BPD‐derived iPSC neurons. miRNA‐34a impairs pre‐ and post‐synaptic development; dentritic branching ↓

iPSC, induced pluripotent stem cells; BPD, bipolar disorder; NPC, neuronal progenitor cell; iNeuron/iN, induced neuron‐like cell.

## Findings suggesting altered synaptic transmission

### Schizophrenia

Alterations of monoamine neurotransmitter systems, most noteworthy DA, play a key role in the pathophysiology of SCZ (Howes *et al*., 2015) and might be a suitable target for cell‐based assays. The DA hypothesis of SCZ was coined over 40 years, ago when the DA depleting properties of reserpine (Carlsson *et al*., 1957) and the DA receptor blocking properties of antipsychotic drugs were linked to improvement of positive symptoms in patients with SCZ (Van Rossum, 1966). Over the years, evidence in favour of disturbed DA transmission as a key feature of the disorder has accumulated. Neuroimaging studies with positron emission tomography (PET) and single‐photon emission computed tomography (SPECT) have identified the pre‐synapse as the most probable origin of DA dysregulation (Howes *et al*., 2012). The cultivation of dopaminergic midbrain neurons thus holds great promise for modelling key pathophysiological traits of SCZ.

There are some findings regarding alterations of the dopaminergic system derived from non‐reprogrammed cells. Utilizing PC12 cells, a cell line derived from rodent adrenal tissue, Kantor *et al*. (2002) succeeded in inducing amphetamine sensitization, a phenomenon endogenously occurring in patients with SCZ (Howes *et al*., 2012). The same group showed protein kinase C (PKC) and mitogen‐activated protein kinase (MAP kinase) to be necessary for amphetamine sensitization to occur (Park *et al*., 2002, 2003) The observation of a biological event thought to be of relevance for DA dysfunction in SCZ in a non‐human, non‐neuronal cell line supports the hope for cell‐based assays to further elucidate alterations of the DA system in SCZ. Nevertheless, impairments of DA transmission could not yet be reliably modelled in reprogrammed neurons derived from patients with SCZ. Hook *et al*. (2014) described a more than 2‐fold elevation in the number of tyrosine hydroxylase (TH) positive (TH+) neurons in cells derived from patients with SCZ compared to those derived from healthy subjects. Moreover, they observed elevated K+‐induced release of DA, epinephrine and norepinephrine in patient‐derived cells. Robicsek *et al*. (2013) observed severely hampered differentiation to dopaminergic and glutamatergic neurons in SCZ‐derived cells. None of the cells expressed the dopamine transporter (DAT). Tyrosine hydroxylase and β3‐tubulin were expressed only at minuscule levels. Dopaminergic neuronal cells displayed altered cell area, neurite number and length. Glutamatergic neurons failed to express T‐box, brain, 1 (Tbr1) alongside with decreased expression of synapsin1 and postsynaptic density protein 95 (PSD‐95) indicating reduced synaptic contacts. A possible explanation for those divergent results may be the different origin of adult somatic cells used in those experiments: While Hook *et al*. reprogrammed skin fibroblasts, Robiscek *et al*. used hair‐follicle keratinocytes. Other variations in the reprogramming procedure, including alternate induction of ventral‐dorsal axis identity of the yielded cell population are discussed in: Hartley *et al*., 2015. Yu *et al*. (2014) describe reduced spontaneous neurotransmitter‐release and spontaneous post‐synaptic currents in SCZ‐derived cells mimicking hippocampal dentate gyrus granule neurons consisting largely of vesicular glutamate transporter 1‐positive (vGLUT1+) cells and a minority of gamma‐aminobutyric acid‐positive (GABA+) cells.

Deficits in depolarization induced vesicle release and impaired synaptic transmission were reported by Wen *et al*. (2014) The investigated cell cultures comprised more than 90% cells expressing vGLUT1 or alpha Ca++/calmodulin‐dependent protein kinase II (α‐CAMKII) neurons derived from two patients suffering from SCZ and major depression from a pedigree carrying a frame shift mutation in *DISC1*. α‐CAMKII has been found to be of relevance for the induction of sensitization to amphetamine (Steinkellner *et al*., 2014). A causative relationship between *DISC1* mutation and synaptic dysfunction was established by generating knock‐in iPSC lines of an unaffected family member and a non‐related control (Wen *et al*., 2014).

Brennand *et al*. (2011) examined a set of five SCZ cases, four of which originated from high‐incidence families. Alongside with the reduced expression of the post‐synaptic marker PSD‐95, which was later found to be attenuated also by Robicsek *et al*. (2013), reduced neuronal connectivity was observed employing a rabies virus assay. Alterations in the expression of genes relevant to glutamate, Wnt signalling and cAMP signalling, were noted by the authors. Reduced synaptic connectivity was rescued by treatment of induced neurons with the antipsychotic loxapine. Loxapine also increased the expression of neuregulin 1 (*NRG1*) and various glutamate receptors (*GRIK1*,* GRM7* and *GRIN2A*), as well as the expression of *ADCY8*,* PRKCA*,* WNT7A* and *TCF4*. No positive effects on connectivity were observed with clozapine, olanzapine, risperidone and thioridazine (Brennand *et al*., 2011).

### Bipolar disorder

In a prospective design, Mertens *et al*. (2015) observed six patients with BPD on Li+ monotherapy. Notably, reduced cellular activity as described by Yu *et al*. was not detected here, but rather an increase in excitability, although identical methods were employed. This indicates a disease‐specific alteration. Cellular hyper‐excitability in BPD‐derived cells was attenuated by Li+ treatment in clinical Li+ responders, but not in Li+ non‐responders. The latter displayed attenuation of electrophysiological alterations by lamotrigine. The authors noted much larger changes in gene‐transcription in Li+ responders when compared to Li+ non‐responders bringing gene expression closer to what is found in healthy subjects. Among the rescued genes, there were 84 directly involved in putative pathological processes, among those PKA/C regulation, neuronal firing rate and Ca++ signalling (Mertens *et al*., 2015).

Li+ was shown to reduce Ca++ transient and wave amplitude significantly more in BPD‐derived neurons than healthy subjects‐derived neurons at 12 weeks post‐differentiation (Chen *et al*., 2014).

## Findings suggesting disturbed neurodevelopment

As iPSC models mimic neuronal development from a very early stage, perturbations of normal developmental steps can be observed on a single‐cell and cell cluster basis.

### Schizophrenia

SCZ is sometimes conceptualized as a neurodevelopmental disorder most commonly manifesting in late adolescence and early adulthood (Keshaban *et al*., 1994; Laurelle, 2000). Gene‐ontology analyses of genes differentially expressed in SCZ and healthy subjects revealed alterations in pathways involved in ‘neurogenesis’, ‘neuronal differentiation’ (Pedrosa *et al*., 2011) and ‘brain development’ (Wen *et al*., 2014; Brennand *et al*., 2015). Two authors describe delayed transformation and maturation of SCZ‐derived cells (Pedrosa *et al*., 2011; Yu *et al*., 2014). Murai *et al*. (2016) found increased microRNA‐219 expression which led to reduced neuronal stem cell proliferation in SCZ patients carrying the *DISC1* mutation. An examination of microRNA expression in patients with SCZ carrying the del(22q.11.2) mutation was conducted by Zhao *et al*. (2015). Thirty‐two microRNAs were significantly upregulated, while 13 were downregulated. Gene‐ontology analysis revealed, among other findings, targets in various clusters important to neurological and psychiatric disease. Yoon *et al*. (2014) observed that single nucleotide polymorphisms (SNPs) in mediators of WAVE signalling (*ACTR2*/*Arp2*) interact epistatically with *CYFIP1* deletion (a gene involved in del(15q11.2)) to increase the risk for SCZ. This epistasis could be mediated by disturbances in apical polarity and adherence junctions in affected neurons. Other authors have observed alterations in gene transcription related to temporo‐spatial cell identity and adhesion (Brennand *et al*., 2011, 2015; Pedrosa *et al*., 2011).

Altered expression of miR‐34 family expression was noted in induced neurons derived from SCZ and schizoaffective (SZA) patients carrying the del(22q11.2) mutation without reaching genome wide significance levels (Zhao *et al*., 2015). Passeri *et al*. (2015) observed a significantly higher transformation efficiency in cells derived from patients carrying the del(16p.11.2) mutation compared to healthy subjects using single‐step transformation in an improved medium.

Abnormalities in cell morphology have been described in cells derived from SCZ patients, however, no clear SCZ‐related phenotype could yet be established. Two studies noted reduced neurite outgrowth (Brennand *et al*., 2011; Robicsek *et al*., 2013). Neurons derived from *DISC1* carriers displayed increased soma size and neurite length 2 weeks after transformation but not 4 weeks after transformation (Wen *et al*., 2014). Cells differentially expressed genes from the gene‐ontology categories ‘brain development’, ‘synaptic transmission’ and ‘dentritic spine’. Topol *et al*. (2015), noticed over‐expression of translation‐related proteins in SCZ‐derived NPCs, but could not detect any abnormalities in cell size that could account for this finding.

Cell motility plays a key role in brain development so that altered cell migration could have neurodevelopmental implications in SCZ. Fan *et al*. (2013) describe markedly increased mobility and reduced adhesion in SCZ‐derived olfactory neurospheres. Furthermore, SCZ‐cells proliferated more readily. Hypermobility was rescued by focal adhesion kinase inhibition. Contradictory to these findings, Brennand *et al*. (2015) observed reduced mobility in human‐induced neuronal SCZ‐cells. In an earlier observation by Pedrosa *et al*. (2011), gene ontology of significantly differently expressed genes in induced SCZ‐neurons revealed ‘cell adhesion’ as an altered functional cluster. In NPCs derived from a patient with SZA, impaired migration, which coincided with preferential expression of a deficient allele of *CNTNAP2* was observed. The patient's not affected father, who carried the same genetic deletion did not display aberrant cell migration while primarily expressing the wild‐type allele (Lee *et al*., 2015). Reduction of NPC migration in SCZ is further corroborated by data from two relatively large cohorts of Topol *et al*. An overall decreased expression of microRNA‐9 in SCZ was driven by a subset of patients below the 25% quantil of healthy subjects. miRNA‐9 was correlated with neurosphere migration, and the phenotype of impaired migration in SCZ was attenuated by over‐expression of miRNA‐9. Marked differences between SCZ and healthy subjects in miRNA‐9 expression could be observed in NPCs and early neurons, but not at 6 weeks after transformation. miRNA‐over‐expression in healthy subjects as well as SCZ‐derived NPCs resulted in a trend towards alterations in the protein expression in pathways related to ‘actin cytoskeleton’, ‘protein localization’ and ‘RNA processing’ (Topol *et al*., 2016).

### Bipolar disorder

A variety of regulatory and neurodevelopmental alterations are thought to play a role in BPD. The review of O'shea & McInnis (2016) gives an overview on neurodevelopmental alterations in BPD with a focus on iPSC models. Madison *et al*. noted significant reduction of cell proliferation in the bipolar offspring of two healthy parents. In four of six iPSC lines derived from BPD, no differentiation to NPCs was achieved. Propagation of NPCs past four or five passages was laborious and could only be achieved for a subset of iPSC lines of BPD. Proliferation of BPD‐derived NPCs was reduced, and paired box protein Pax‐6 (PAX6) expression decreased. Gene expression did not significantly differ between BPD and healthy subjects derived fibroblasts and iPSCs, however, in NPCs, 18 genes showed > 1.5 fold difference in expression. Multiple genes involved in spatial identity regulation were altered (*NKX6‐1*,* LEF1*,* NEUROG1*,* NRG3* and *SPARCL1*; Madison *et al*., 2015).

Impairment of ventral‐dorsal patterning and spatial identity was described by Chen *et al*. (2014) who observed that cells derived from BPD patients display increased *DICER1*,* NKX2‐1* and *PAX2* expression (all of which promote ventral cell fate) and reduced expression of *EMX2* and *HOXA1,6* and *7* (which promote dorsal cell fate). Li+ reportedly up‐regulates Wnt signalling (Valvezan & Klein, 2012), thus dorsalizing early NPCs (Wang *et al*., 2014). In cerebellum slices of Li+‐free BPD patients, significantly elevated levels of miR‐34a were detected. In BPD iPSC‐derived neuronal cells, inhibitory effects of miR‐34a on the expression of *ANK3* and *CACNB3* were described together with derogation of pre‐ and post‐synaptic development such as reduced dentritic branching (Bavamian *et al*., 2015). In non‐neuronal cells, miR‐34a was earlier described as an inhibitor of Wnt signalling (Hashimi *et al*., 2009).

Using a high‐throughput label‐free imaging platform (BIND scanner) in BPD, Wang *et al*. (2014) could detect significantly higher peak wave length in cells derived from Li+ responders when compared to Li+ non‐responders. This suggests greater adhesion of cells derived from Li+ responders when compared to non‐responders. None of the other parameters (cell count, cell fraction, and cell perimeter) were significantly different between healthy subjects and BPD patients and Li+ responders and non‐responders.

## Findings on alterations in energy metabolism

### Schizophrenia

Alterations of mitochondrial function metabolic stress are thought to contribute to the development of SCZ (Rajasekaran *et al*., 2015). Irrespective of treatment history, patients with SCZ differed from healthy subjects in a micro‐array analysis of genes relevant to energy metabolism and oxidative stress. In a proteomics‐analysis, about half of the significantly altered proteins were related to energy metabolism and oxidative stress (Prabakaran *et al*., 2004). Employing non‐reprogrammed SCZ‐derived fibroblasts, Fourier *et al*. observed altered cell metabolism in response to oxidative stress in a collective of first‐episode SCZ patients. Significant changes were observed in arginine‐, extracellular matrix‐ and collagen‐related pathways. Fatty acid metabolism and neurotransmitter‐related metabolism were unaltered (Fournier *et al*., 2014). Alterations in energy metabolism and reactive oxygen species (ROS) have been observed on a genetic, morphological and functional level in human‐induced neuronal cell models of SCZ. Paulsen *et al*. did not note any alterations in ATP‐coupled respiration, but rather increased extra‐mitochondrial O_2_ consumption and elevated levels of reactive oxygen species (ROS). These changes occurred in SCZ‐derived NPCs but not in iPSCs or fibroblasts. ROS‐levels were rescued by treatment with valproate (Paulsen Bda *et al*., 2012). In another study, the same group observed elevated levels of potassium and zinc levels in SCZ‐derived NPCs again rescued by valproic acid treatment (Paulsen Bda *et al*., 2014). Increased levels of ROS and concomitantly decreased mitochondrial membrane potentials and DNA damage suggesting increased apoptosis were measured by Brennand *et al*. (2015) In the same sample, cell‐to‐cell variability but not overall expression of 70 kd heat‐shock protein (HSP70) expression after oxidative stress was significantly elevated in SCZ. This finding is in line with increased variability, but no changes in mean expression of HSP70 expression following ethanol and methyl mercury stimuli in SCZ‐derived NPCs observed by Hashimoto‐Torii *et al*. (2014). In experiments performed by Robiscek *et al*., keratinocytes derived from healthy subjects displayed baseline differences in O_2_ consumption as compared to keratinocytes derived from patients with SCZ, while their iPSCs did not differ in this regard. Inhibition of mitochondrial complex‐one driven respiration by DA was significantly elevated in keratinocytes, as well as iPSCs and NPCs, derived from SCZ patients. Alterations of mitochondrial network connectivity and distribution of mitochondria were described in SCZ patients (Robicsek *et al*., 2013). Batalla *et al*. correlated neuronal glutamate‐glutamine (GLX) and *N*‐acetylaspartate (NAA) with apoptotic markers in non‐reprogrammed fibroblasts derived from first‐episode SCZ patients in a combined cell culture and proton magnetic resonance spectroscopy approach. The authors could establish a relationship between decreased GLX and NAA levels in the anterior cingulate and condensed chromatine in staurosporine‐treated fibroblasts suggestive of excitotoxicity in the brain (Batalla *et al*., 2015).

In an approach integrating induced cell models and functional magnetic resonance imaging (fMRI), D'Aiuto *et al*. (2015) investigated possible relationships between latent Herpes simplex virus 1 infection and cognitive parameters in healthy subjects and SCZ using induced neurons as a model for possible contributions of infectious diseases to the pathogenesis of SCZ. However, induced neurons and fMRI data were not derived from the same patients in this study.

### Bipolar disorder

BPD‐derived neurons displayed a peculiar phenotype of overall smaller mitochondria that showed greater function in the JC‐1 ‐ mitochondrial membrane potential assay when compared to healthy subjects. Li+ treatment rescued mitochondrial size but did not significantly alter membrane potential (Mertens *et al*., 2015).

## Conclusions

Most studies investigating patients with SCZ were aiming to observe an aetiologically homogeneous population, focusing on high‐incidence families and patients with high impact gene variants, which is a sensible approach for studying subtle alterations of genetic perturbations, possibly giving cues for general mechanisms underlying this complex polygenetic and poly‐aetiologic disorder (Marchetto & Gage, 2014). Identification of alterations in well‐defined cases may facilitate the examination of larger, more heterogenous populations.

The examination of iPSC‐derived DA neurons from a pair of monozygotic twins discordant for Parkinson's disease, while carrying a distinct predisposing mutation, yielded significantly different findings in monoamine oxidase B (MAO‐B) activity and DA availability (Woodard *et al*., 2014). This can be interpreted as proof of concept, suggesting that also non‐genetic effects may be observed in reprogrammed cells. The method may thus allow for the identification of biological correlates of environmental contributions to complex disorders, in particular in phenotype‐discordant monozygotic twins. As the majority of clinical cases are sporadic rather than monogenetic or familial, many aspects may, however, be overlooked when restricting research to twins discordant for a certain disorders. Attempts to recruit patients with distinct clinical parameters have been made. In particular, observing patients with distinct reaction patterns to medication, such as clozapine resistance (Paulsen Bda *et al*., 2012) or Li+ responsiveness (Chen *et al*., 2014; Wang *et al*., 2014) might be interesting.

In any case, for reprogrammed cells to hold their promise of modelling ‘brain disease in a dish’ (Marchetto & Gage, 2012) and start playing a prominent role in testing and development of medicinal compounds or pathogenetic studies, more work needs to be done. There are some findings discussed earlier, where loxapine (Brennand *et al*., 2011), valproic acid (Paulsen Bda *et al*., 2012, 2014), or Li+ (Mertens *et al*., 2015) could revert alterations observed in cells derived from patients with SCZ or BPD. For the time being, it is unclear whether any of these observations would be predictive of clinical efficacy of a compound, or if the observed alterations translate to pathologies *in vivo*. There are still plenty of pieces missing in the puzzle of modelling psychiatric disorders *in vitro*. To be able to interpret findings from reprogrammed cell assays, a robust link between clinical traits and observations in cell culture systems has to be established. In order to achieve comparability between studies, meticulous description of the reprogrammed cells’ phenotype and expression patterns is paramount. As elucidated by the example of discrepant findings between the reprogramming of adult cells from patients with SCZ to dopaminergic neurons (Robicsek *et al*., 2013; Hook *et al*., 2014) mentioned earlier, a finding or lack thereof might not be interpretable without thorough characterization of the cell. As neither TH nor DAT are truly unique to dopaminergic midbrain neurons (Ciliax *et al*., 1999), a whole battery of tests is necessary to pinpoint cellular identity. Electrophysiological behaviour, tempo‐spatial identity, gene expression, characteristic proteins, pharmacological sensitivity and morphology should, where appropriate, be used to confirm comparability of findings achieved with different reprogramming techniques. Reprogramming remains a volatile procedure, where small alterations in the protocol might change the outcome. In the case of single‐step reprogramming to DA neurons, it has been shown that dish surface characteristics influence reprogramming efficiency and cell morphology (Yoo *et al*., 2015). While it may be impossible to ascertain in how far a reprogrammed cell represents an *in vivo* counterpart, thorough characterization of its characteristics will help to increase faith in the validity of the model (Srikanth & Young‐Pearse, 2014; Hartley *et al*., 2015).

Data from cell cultures and genetic analysis will need to be integrated with imaging studies, specifically PET and fMRI, to create a more comprehensive picture of psychiatric disorders (Brennand & Gage, 2011). To the best of our knowledge, only two publications combine imaging data and cell culture findings trying to crosslink information in a meaningful way. The study of D'Aiuto *et al*. (2015) primarily employed cell culture techniques to prove the concept of latent HSV‐1 infection in human neuronal cells. Batalla *et al*. (2015) did not utilize reprogrammed cells, but attempted to directly correlate *in vitro* with *in vivo* data. Surprisingly, strong correlations between brain metabolites and behaviour of cultured cells were found. Establishing correlations between single‐cell phenomena, clinical features and imaging data in individual patients may facilitate the interpretation of *in vitro* findings in the future. Many features of cultured cells can be altered by the reprogramming process or remnants thereof, as well as by non‐physiological circumstances such as the presence of immature cells in the culture, lack of synaptic contacts and glia, or loss or disturbance of the epigenetic imprint. Furthermore, cell culture and imaging techniques yield profoundly different data. For instance, increased cell size *in vitro* does not necessarily correspond to altered volumes in MRI studies. When going beyond describing differences between healthy subjects and patients, clear hypotheses regarding pathophysiological phenomena likely to occur on a single‐cell basis may be helpful. PET in particular could establish a link between *in vivo* and *in vitro* observations by correlating factors such as receptor availability or neuro‐transmitter release with genetic and cell culture data. Unlike fMRI, which is limited to observations on a network level, PET is able to visualize targets on a molecular scale. Once cellular and clinical phenotypes have been put into relation, it may be possible to predict clinical outcomes, such as response to medication, based on cell culture models.

If only a specific phenomenon or marker is of interest rather than the process of development and maturation *per se*, it would be desirable to identify just the right cell type suited for the assay, and to convert cells in a single‐step method. This way, the sample size can be increased allowing for the recruitment of a more realistic study population without sacrificing too much statistical power. However, the role of cell maturity and possible effects of the source cells’ epigenetic imprint will have to be considered.

A recent meta‐analysis of PET imaging data on the DA system in patients with SCZ has yielded large effect sizes for results indicating a pre‐synaptic dysfunction of the DA system in SCZ (Howes *et al*., 2012). There are well‐established imaging procedures for quantifying DA release and synthesis *in vivo,* as well as reliable reprogramming techniques yielding functional dopaminergic midbrain neurons of high purity. The pre‐synaptic disturbance of the dopaminergic system therefore seems to be an accessible and promising target for further experiments. As antipsychotic drugs commonly interfere with dopaminergic transmission by blocking post‐synaptic D_2/3_ receptors, establishing an *in vitro* model of pre‐synaptic DA dysfunction in SCZ might also facilitate drug development or pre‐clinical testing of antipsychotic compounds in the future.

On the other hand, many research questions regarding early brain development in SCZ, the development of psychosis, and brain alterations in subjects who are at risk mental state for psychosis remain unanswered. In the future, more complex traits, in particular neuro‐developmental perturbations, could be successfully modelled in organoids (Marchetto & Gage, 2014). Brain organoids comprise multiple neuronal and glial cell types, resembling the brain in early pregnancy (Lancaster *et al*., 2013; Lancaster & Knoblich, 2014). As of today, development of brain organoids beyond an early stage is hindered by the lack of vascularization, so that later developmental stages remain inaccessible. Some events predisposing to the outbreak of SCZ, such as intra‐uterine infection, birth trauma or seasonality of birth occur during pregnancy and might to some extent be modelled *in vitro*. While some proportion of the genetic loci increasing the risk for SCZ are assumed to impact early brain development, others might not contribute to the appearance of the phenotype until much later in life.

## Techniques of cell reprogramming

The process of reprogramming an adult somatic cell is dependent on multiple factors each potentially influencing the induced cells’ phenotype and function. Until now, multiple cell types of different origin have been successfully reprogrammed. However, as of yet, no cell type has been shown to be clearly superior. Even while comparatively difficult to harvest, skin fibroblasts remain the most common source, possibly because there are many protocols readily available in the literature. Despite their individual advantages, alternative, cell types such as CD34+ blood cells, squamous cells derived from urine, or hair follicle keratinocytes are as of yet rarely used. The properties, advantages and disadvantages of various cell types are reviewed by (Raab *et al*., 2014). The impact of the original cells is not confined to traits such as reprogramming efficiency, availability or number of published methods, but stretches into the realm of epigenetics. DNA and histone methylation patterns remain partly that of the tissue of origin, so that iPSCs differentiate most readily to closely related cells (Kim *et al*., 2011; Vaskova *et al*., 2013). One recent examination did, however, come to the conclusion that genetic and epigenetic constitution of the donor outweigh cell‐type effects (Kyttälä *et al*., 2016). By treatment with chromatin‐modifying compounds or secondary and tertiary reprogramming, the differentiation‐inhibiting properties of epigenetic memory can be attenuated (Kim *et al*., 2010). However, as a cell's epigenetic imprint might influence a disease‐relevant phenotype, such procedures remain controversial.

In order to successfully reprogram adult somatic cells, either to a different cell type or stem cells, the selected transcription factors must be delivered to the cell. Multiple vectors have been reported so far, each bearing benefits and disadvantages, so that it remains an individual choice which system to employ for a specific problem. For a comprehensive analysis of reprogramming vectors, see (Hu, 2014). In addition to high reprogramming efficiency, the ability to enter the desired cell, safety, price and the dynamics of transgene silencing are key points of interest for vectors in research. As there are at the time being no ambitions for the development of cell therapies based on reprogrammed cells in psychiatry, the issues of oncogenic transcriptional factors as well as immunogenicity of many vectors and, to a lesser extent, the mutagenicity of integrating vectors are of limited concern.

In most studies considered for this review, patient‐derived cells were first reprogrammed to iPSCs and later differentiated to NPCs and/or any neuronal population. Today, there are various published methods for the generation of patient‐derived neurons either through reprogramming to iPSCs, or through direct transformation of adult cells to functional neuronal cells. (Tran *et al*., 2013) Depending on the research issue, either method has its merits. While iPSCs offer the possibility to observe developmental steps as they would occur *in utero*, single‐step reprogramming techniques have the advantage of being less costly and laborious. Although directly converted cells reach a mature phenotype much faster than iPSC‐derived cells (Caiazzo *et al*., 2011; Kriks *et al*., 2011), it remains unresolved whether this is a benefit allowing for rapid observation of functional differences, or whether it is a limitation, possibly omitting pathological steps in development while keeping much of the epigenetic imprint of the tissue of origin.

Today, a multitude of reprogramming techniques exists. First, direct conversion of fibroblasts to dopaminergic midbrain neurons by forced expression of the factors published by Vierbuchen *et al*. (2010) (*Ascl1*,* Brn2* and *Myt1 l*) together with *Lmx1a* and *FoxA2* (Pfisterer *et al*., 2011), or solely *Mash1*,* Nurr1* and *Lmx1a* (Caiazzo *et al*., 2011) was reported. As of yet, a variety of protocols for the generation of specific neuronal populations have been published. Second, starting from protocols promoting neural induction and functional floor plate formation using human embryonic stem (hES) cells (Chambers *et al*., 2009; Fasano *et al*., 2010). Kriks *et al*. (2011) developed a method for generating dopaminergic neurons with marker profile and distinct electrophysiological features reminiscent of those peculiar of human midbrain development. In this case, the required co‐expression of *FoxA2* and *LMX1* typical of mature dopaminergic neurons is obtained by combining inhibitors of the transcription factors SMAD, sonic hedgehog agonists and *GSK3B* inhibition‐mediated Wnt signalling activation. Along with the optimization of methods to produce 2D cultures representative of specific types of neurons, it is worth mentioning the recent efforts made to generate 3D neuronal model systems that have proven useful to model neuro‐developmental diseases (Lancaster *et al*., 2013; Mariani *et al*., 2015; Qian *et al*., 2016). In particular, developing forebrain cerebral organoids showing significant overlap between differentially expressed genes and SCZ risk genes, as well as midbrain cerebral organoids specifically expressing dopaminergic neuron markers were recently obtained from iPSCs (Qian *et al*., 2016). Although comprehensive physiological characterization is not yet available, midbrain organoids expressing dopaminergic neuron markers are generated starting from previously successful protocols used to model Parkinson's disease (Kriks *et al*., 2011) and show a relative early appearance of mature TH‐expressing neurons. Considering the cell mass‐fold expansion typical of 3D organoids, such a protocol would have the advantage of yielding high numbers of neurons in a cost‐effective way and relatively short time, representing a valid alternative to 2D cultures. However, a limitation of the system might be the poorly defined identity of the cellular population obtained by this method: At day 65 only about half of the *FoxA2* ‐expressing neurons were also showing TH expression. While obviously not suitable for certain applications, like transplantation in animal models, the self‐organization observed in such organoids might reflect a more reliable approximation of the early steps in the maturation of the developing midbrain and thus represent an advantage in *in vitro* studies, e.g. when the role of mutations on proper neuronal maturation is hypothesized.

Cell maturity is likely to play an important role in the interpretation of data acquired from induced human neuronal cells. While cells derived from older individuals are reprogrammed at a lower efficiency than cells derived from younger individuals, reprogramming to an iPSC state seems to rejuvenate the cell (Li *et al*., 2009; Mahmoudi & Brunet, 2012). The relevance of cell donor age is however questioned, as other groups reported efficient reprogramming of fibroblasts from elderly individuals (Marchetto *et al*., 2010). Furthermore, an increase in iPSCs reprogramming efficiency appears to be achievable from both, high fibroblast passage number and aged donors, respectively, through inhibition of the upregulated cell cycle factor p21, potentially expanding sample typology (Trokovic *et al*., 2015).

Another point worth discussing are limitations of modelling SCZ through reprogrammed neurons: a population of fibroblast‐derived early neurons at 6 weeks differentiation and neuronal progenitor cells derived from patients with SCZ and healthy subjects resembled first trimester neuronal cells regarding gene‐expression patterns (Brennand *et al*., 2015). The onset of psychotic illnesses such as SCZ and BPD is most common in late adolescence and early adulthood, however, a proportion of patients will develop first symptoms in infancy or much later in life (Kessler *et al*., 2005; Jones, 2013). Even though SCZ is associated with subtle unspecific deficits in social functioning, motor skills and cognition before a prodromal phase occurs (Murray *et al*., 2006), and even though some traits like anxiety, attention‐deficit and subclinical changes in mood can predate BPD by many years (Serra *et al*., 2015), those alterations do not occur in all individuals and are not necessarily causally linked to the development of psychosis. Immature cells resembling cell populations found in the prenatal age can most likely be employed to model alterations of cellular functioning very early in the development of those disorders, as discussed above. However, they do not necessarily reflect pathological mechanisms occurring later in life. As an example, considering the role attributed to age as a central aetiological factor in the field of neurodegenerative diseases, progerin treatment has been shown to substantially accelerate the process of maturation and degeneration in iPSCs, resulting in a good model for Parkinson's disease (Miller *et al*., 2013). Having this in mind, additional cellular model systems should be explored to mimic the effects of age‐related effects on the onset of psychotic disorders.
